# Hiding in Plain Sight: Conceptualizing the Creeping Crisis

**DOI:** 10.1002/rhc3.12193

**Published:** 2020-04-12

**Authors:** Arjen Boin, Magnus Ekengren, Mark Rhinard

**Keywords:** creeping crises, transboundary crises, crisis management

## Abstract

The COVID‐19 crisis is a stark reminder that modern society is vulnerable to a special species of trouble: the creeping crisis. The creeping crisis poses a deep challenge to both academics and practitioners. In the crisis literature, it remains ill‐defined and understudied. It is even harder to manage. As a threat, it carries a potential for societal disruption—but that potential is not fully understood. An accumulation of these creeping crises can erode public trust in institutions. This paper proposes a definition of a creeping crisis, formulates research questions, and identifies the most relevant theoretical approaches. It provides the building blocks for the systematic study of creeping crises.

## 摘要

2019新型冠状病毒(*COVID‐19*)是一个严峻提示: 现代社会在慢性危机这一特殊困难面前是脆弱的。慢性危机为学术界和从业人员都造成了一个巨大挑战。在相关文献中, 慢性危机的定义并不清晰且缺乏研究。管理慢性危机更为困难。作为一种威胁, 它具备扰乱社会的潜能, 但这种潜能目前没有被完全理解。这类慢性危机的累积能削弱公众对政府机构的信任。本文提出了对慢性危机的定义, 制定了研究问题, 并识别了最为相关的理论措施。本文为有关慢性危机的系统性研究提供了基础。

## Resumen

La crisis de COVID‐19 es un claro recordatorio de que la sociedad moderna es vulnerable a una especie especial de problemas: la crisis progresiva. La progresiva crisis plantea un profundo desafío tanto para los académicos como para los profesionales. En la literatura, permanece mal definida y poco estudiada. Es aún más difícil de manejar. Como amenaza, conlleva un potencial de disrupción social, pero ese potencial no se comprende completamente. La acumulación de estas crisis progresivas puede erosionar la confianza pública en las instituciones. Este artículo propone una definición de crisis progresiva, formula preguntas de investigación e identifica los enfoques teóricos más relevantes. Proporciona los componentes básicos para el estudio sistemático de las crisis progresivas.

## Introducing the Creeping Crisis^1^

In December 2019, a new Coronavirus emerged in China. As little was known about the immediate consequences of the virus, the world paid scant attention. That hardly changed when China announced that the outbreak of the virus was dangerous and subsequently locked down its entire population, bringing its juggernaut economy to a sudden halt. When the first cases emerged in one European country, other countries did not take any measures. When the World Health Organization branded Europe as the new hot spot of the pandemic, the United States did not react. When the first deaths were registered on the U.S. West Coast, the New York City mayor admonished his citizens to stick with their routines (keep going to restaurants!).

The COVID‐19 crisis crept up on countries, cities, and hospitals. It arrived in full view, yet still surprised politicians, hospital administrators, pundits, business owners, and citizens. But the COVID‐19 pandemic is not the first crisis to arrive creeping and causing devastating surprise.

In the 1980s, an unknown disease hit vulnerable Americans—homosexuals, minorities, drug users—particularly hard. Many people were dying of what would eventually be known as Acquired Immune Deficiency Syndrome (AIDS). For years, politicians did not speak of the disease—let alone address it. It was not until Rock Hudson, the iconic movie star, died of AIDS (in 1985) that the Reagan administration began to define the AIDS epidemic as a full‐blown health crisis. The AIDS crisis would continue unabated for years, playing itself out in slow motion, until it was eventually brought under control (in the Western world, at least). Looking back, one might wonder how many lives could have been saved if this devastating disease had been recognized as a crisis much earlier (Guillén & Perrow, 1990).

In the summer of 2015, Europe slowly woke up to a migration crisis. Thousands of migrants, from all over the world, were reaching the Greek islands every day. They were on their way to the rich European countries—Germany, Sweden, the United Kingdom—where a better life was awaiting them, or so they believed. Migration flows to Europe were nothing new, of course. For many years, people from desperate regions had tried their luck and entered Spain and Italy, risking their lives crossing the Mediterranean (many people died trying). It was not until thousands of refugees flooded the Greek islands, then the train stations of Budapest and Vienna, that European politicians defined the migration flows as a crisis. That did not stop the crisis: it would take a long time before European countries found a way to bring the crisis under control. And nobody is under the illusion that the crisis has been solved.

On 18 September 2019, the Dutch lawyer Derk Wiersum was assassinated in front of his Amsterdam house. Wiersum was representing a crown witness against the leader of a feared criminal gang. The assassination marked a turning point in the common perception of organized crime in the Netherlands. For years, experts had warned that Dutch criminals were major players in the worldwide drug production and trade of chemical drugs. They had warned against the perverse influence of underworld money on the integrity of public institutions. They pointed to the ever‐hardening violence—including a beheading, public executions, and the killing of innocent bystanders—that marked the ongoing gang wars. Yet, politicians had not made organized crime a policy priority. The day after the murder, politicians professed to be shocked and called for an integrative approach to target transboundary crime.

Modern society is beset by a special species of trouble that is hard to define and even harder to manage (see Table 1). It is a species of trouble that is easy to ignore. It is the threat that lurks in the background, manifesting itself through a series of seemingly manageable incidents (Beamish, 2002). The damage and sorrow it causes is hidden or localized. Its symptoms may be addressed, but the underlying causes escape serious treatment. It is the threat that may attract the worried attention of certain experts and certain politicians, but fails to generate a sense of urgency among a wider public. As a threat, it carries the potential for societal disruption—but that potential is not fully understood or actively downplayed. When left festering, the consequences can be disastrous.

**Table 1:** Examples of Creeping Crises

The accumulation of civil unrest in North Africa (a gradual trend generated by deepening of economic inequality, political conflict in neighboring lands, and environmental degradation), which has different manifestations at different times and degrees of severity: boats overloaded with immigrants crossing the Mediterranean, political uprising in Syria, flows of people reaching the Balkans, groups of people camping out near Calais.
The accumulation of personal data in the hands of private companies and governments (a gradual trend fed by people's reliance on smart devices, low levels of regulation, demands for convenience), which leads to different episodes at different times that vary in severity: the leaking of personal data, the hacking of AI controls in automobiles, the combing of private data by intelligence agencies, declining trust in national regulators.
The overprescription of antibiotics has built up a general resistance of some bacteria (a gradual trend taking place over many years, owing to a combination of declining health care systems in the developing world that use antibiotics as a “cure all”, improper prescriptions in the developed world, and the mutation and strengthening of certain bacteria). This leads to a variety of diffuse problems such as superbugs in hospitals.
The interconnectedness of global communication and the growing use of social media (a gradual trend emerging over the past 20 years, owing to IT innovation and the need to monetarize the news media), which has led to media fragmentation, echo‐chamber dialogues, and exploitation by troll farms in the form of disinformation campaigns. These specific problems have emerged at different points in the last few years, with different degrees of severity and threat assumptions.
Climate change has had a myriad of different effects, emerging at different moments and with different degrees of severity. Strangely warm summers, forest fires in Sweden, submerged islands in the Indian ocean, and declining insect populations are all diffuse “small bangs,”​​​ which emanate from a central problem.

What is perhaps most worrying about the creeping crisis, however, is its potential to undercut the legitimacy of public institutions. Today, people worry not *if* but *when* global warming, migration, returning IS‐warriors, disruptive technologies, market disequilibrium, or income inequality will cause a major crisis. Experts have predicted, for instance, the emergence of a new pandemic for years (“it's long overdue”). When COVID‐19 escalated into public view, its long trajectory could be construed as willing ignorance on the part of those public institutions that were designed to protect citizens.

The causes may remain unclear, even in hindsight, but many actors will receive at least part of the blame. A long incubation phase makes it hard to argue that “they could not see it coming” or that they did not have time to act. A stuttering defense undermines public faith in leaders. It also undermines faith in the institutions that should have played a pivotal part in preventing, detecting, and managing the creeping crisis. Creeping crises thus portend a “slow drip” delegitimization of public institutions. This threatens the response capacity needed for effective crisis leadership and governance arrangements.

### Towards an Understanding of the Creeping Crisis

The creeping crisis poses real conceptual problems for academics. Whereas the “traditional” crisis can be more or less neatly delineated, it is not clear at all how these simmering or creeping crises can be precisely described and reliably recognized. How to recognize the crisis potential of an emerging threat? How does a creeping crisis differ from a protracted societal problem or what Peters (2015) refers to as a “super wicked problem”? When does a creeping crisis become a “real” crisis? When does a “real” crisis become a creeping crisis? When does a creeping crisis end?

Creeping crises also pose real challenges for the world of practice (Boin & Lodge, 2019). Practitioners often find it hard to recognize the devastating potential of these creeping crises. In some cases, they do not seem to recognize the creeping crisis at all (manifestation of the creeping crisis is met with shocked surprise). In other cases, practitioners address what in hindsight turn out to be mere symptoms, allowing the bubbling crisis to develop its devastating potential under the radar. Even after tipping into a full‐blown crisis, practitioners may discover that easy solutions are not available and a protracted battle lies ahead. As the crisis plays out in slow motion, practitioners find themselves dealing with one nasty surprise after another. The financial crisis and COVID‐19 pandemic came creeping and kept returning in yet another guise, exhausting crisis managers, politicians, and astounded citizens (Bernanke, Geithner, & Paulson, 2019).

We need to understand the creeping crisis: how and why it can develop in full view, seemingly undisturbed, building steam until it is too late. In this article, we seek to define the creeping crisis, identify the challenges that it poses to academics and practitioners alike, formulate research questions, and select useful theoretical frameworks that may help address these questions.

## Conceptualizing the Creeping Crisis

Crisis researchers have long recognized a difference between two theoretical ideal types: the “fast‐burning” and “slow‐burning” crises (‘t Hart & Boin, 2001; see Table 2). Crisis research has mostly focused on the fast‐burning kind. The fast‐burning crisis explodes on the scene and quickly disappears into the history books after it is brought under control. This crisis is a discrete event, an exceptional situation with a clear beginning and end. It matches a popular definition of crisis as a widely recognized threat that requires an urgent response under conditions of deep uncertainty (Rosenthal, Charles, & ‘t Hart, 1989). The Cuba Missile Crisis was the quintessential example for political scientists (Allison, 1971). Today it probably is 9/11 (at least for American political scientists).

**Table 2:** Crisis Typology Based on Temporal Dimensions

		*Closure*	
		FAST	SLOW
	FAST	Fast‐burning	Long‐shadow
*Onset*			
	SLOW	Cathartic	Slow‐burning (or creeping crisis)

*Source*: ‘t Hart and Boin (2001).

The slow‐burning or creeping crisis has a long incubation time and may keep simmering long after the “hot phase” of the crisis is over. It does not have a clear beginning or ending. It can remain undefined for a long time; it can change meaning during its lifespan. Tensions between Iran and the United States, refugee flows, the changing climate, wildfires in Australia and, of course, the COVID‐19 pandemic—these are all slow‐burning crises: they have a seemingly permanent, epochal character, generating regular outbursts without reaching closure.

The creeping crisis has a temporal and a spatial dimension. Threats evolve as slumbering processes over longer periods of time, with unclear beginnings and ends (global warming), suddenly manifesting themselves as seemingly isolated “explosions” (forest fires) (Ekström, 2016). From a temporal perspective, they can be qualified as “slow‐burning” with incidental “sparking.” From a spatial perspective, threats increasingly appear as the outcome of transboundary processes that start in distant lands (the Syrian war) or seemingly unrelated policy domains before they “explode” into a regional or local crisis (border management crisis in Europe 2015). One might say these threats are “local” (Robertson, 1995) or even “translocal” (Ekengren, 2018) in nature. These crises should not be analyzed as exceptional situations delimited in time and space, but as permanent global threats that can manifest themselves as seemingly acute crises at the local level (George, 1991).

Crisis researchers have not paid much attention to the creeping crisis. Two developments have begun to shift the focus. First, scholars have begun to trace the roots of these discrete events and quickly discovered that most of these crises were the result of a *process*, alternatively described in terms of incubation, escalation, and root causes. To understand the origins of crisis, it is now realized that a long‐term view is necessary. Second, scholars have noted that many societal problems appear to have crisis characteristics—even if they are not described in terms of crisis or being treated as such (cf. Erikson, 1994). These scholars have pointed to conditions that present real dangers to societal values, the functioning of critical infrastructures, or the safety of people. In short, the focus has shifted. Now we need a proper definition to start systematic research.

### Starting Point: The Crisis Definition

A good starting point is the “regular” crisis definition offered by Rosenthal et al. (1989). They speak of a crisis when political‐administrative elites perceive a threat to the core values of a society and/or life‐sustaining systems in that society that must be addressed urgently under conditions of deep uncertainty (cf. Rosenthal, Boin, & Comfort, 2001). Critically, this definition recognizes that threat and urgency are *social constructions*. In their perspective, crises do not exist independently of people's perceptions. Crisis, then, is the label that observers like us attach to the shared sense among a group of people that something they value is under threat. Their sense of threat is deepened by a feeling of uncertainty (about causes, dynamics, effects, and possible solutions). But it is only a crisis when people perceive something as an *urgent* threat—something that must be addressed *now*.

How do people collectively come to share a sense of crisis? Many factors undoubtedly play a role (e.g., collective fear, media dynamics, and political calculations), but the question is when and how, exactly, the perceptions of many individuals begin to converge. Some threats create instant convergence of perceptions. An explosion, a large earthquake, an epidemic—such events do not leave much room for differing interpretations. But many, perhaps most, threat situations are not self‐evident. People must grow convinced that a certain development or condition threatens core values, public institutions, critical infrastructures, or the direct well‐being of people.

This brings us to an important insight. *A collective perception of threat may arise organically or it might be constructed. Either way, it is a social process that plays out over time. That process may unfold instantaneously; it can also take years*.

An alternative, and complementary, definition views crisis as an empirical phenomenon—a real threat—that has the potential to cause serious damage to critical values or systems. This definition is *objective* in nature. Examples include a hurricane, a flood, a cyber attack or a wildfire—distinct phenomena that are measurable, its effects observable.

In this objective definition, the perception of this or that group is irrelevant. This type of definition is dominant in the disaster and critical infrastructure literatures. Whether there is an impact of these natural and technological threats is rarely a matter of deep discussion. What counts is how you measure that impact. In this line of thinking, the *development* of threats attracts much interest. An interesting question, for instance, is how and why complex and tightly coupled systems produce certain types of threats (Boin, 2019; Perrow, 1984). If causes and mechanisms of escalation are known, then perhaps points of intervention can be recognized and the crisis may be halted before it is too late.

### Towards a Definition of Creeping Crisis

In our perspective on the creeping crisis, we make use of both the subjective and the objective perspective. The subjective crisis definition emphasizes the importance of *attention*: if political elites, media, and the public do not collectively share a sense of crisis, it is hard to speak of a crisis in this perspective. The objective definition emphasizes the importance of *accumulation of threat potential*. In this objective perspective, a crisis is best understood as a developmental process with root causes, an incubation phase, an acute phase, and an aftermath.^2^

What sets the creeping crisis apart from other types of undesirable events is the temporal dimension. *Both* the attention and the actual threat potential develop over time. The “creeping” refers to the slow speed of development, when compared to other types of events. It can be described in terms of evolving disruptions that may be detectable but are hard to agree on.

A key characteristic of the creeping crisis is the *absence* of attention (where a crisis is defined by a high level of attention). The damage potential of a threat may grow, but it matters in this definition whether different segments of society label the growing threat as a crisis. There may be a tipping point, which marks the threshold that must be passed for the crisis to attract sufficient social and political attention so that it is *experienced* as a crisis. The tipping point is not necessarily a moment of eruption after which the crisis quickly fades—the creeping crisis may keep on creeping. Both the beginning and the ending of these creeping crises are blurry.

These initial insights bring us to a working definition:

*A creeping crisis is a threat to widely shared societal values or life‐sustaining systems that evolves over time and space, is foreshadowed by precursor events, subject to varying degrees of political and/or societal attention, and impartially or insufficiently addressed by authorities*.

This definition brings together the objective and subjective perspectives on crisis. It references the gradual emergence and development of a threat to core values, democratic institutions, critical infrastructures, the environment, and the well‐being of people; it also acknowledges the importance of attention that is required to initiate and sustain remedial action.

Our working definition suggests that these crisis types may exist for a considerable amount of time (months, years) without being formally recognized. Paradoxically, perhaps, this definition gives rise to a sense of optimism as it recognizes a window of opportunity for authorities to address the threat before it explodes into view. The creeping crisis provides authorities with a precious commodity: time to act.

But here lies the rub. A generous time slot will not make a difference if authorities do not realize that time is of the essence. Yet, as our definition implies, there usually is a high level of uncertainty with regard to the actual status of the threat and variation in the level of concern that it evokes among different stakeholders. Manifestations of the emerging threat tend to be diffuse. Symptoms appear occasionally, seemingly spontaneously, in various forms. Key actors, however, may not immediately recognize and immediately agree that these manifestations are symptoms of an underlying, much more serious problem. By effectively addressing a symptom in the early developmental phase, attention for the threat may dissipate, which allows the threat to regain momentum under the radar.

Authorities may simply ignore, even deny the symptoms. Even when political attention finally reaches a tipping point (the crisis is declared), the creeping crisis may still proceed in slow motion (the patient gradually declines). The creeping crisis will thus require *sustained attention* of authorities if it is to be brought under control.

## Questions for Research

The two key variables that emerge from the previous discussion are *attention* and *development*. To start with the latter, the pace of crisis development can vary between slow and fast. The pace may vary during the lifespan of a crisis: it may come creeping, accelerate and erupt, and then take a long time to fade. It may come creeping and burn fast. Attention can show even more variation. Not only does the intensity of attention ebb and flow, but attention can reach different levels with different audiences at different times. Citizens may be worried about an emerging threat while politicians ignore or dismiss it; rising media attention may force politicians to do something; a symbolic intervention may quiet the minds of some worried citizens, but spike interest in others. In short, attention may spark and fade in different ways among a wide set of actors.

These two variables helped us formulate a set of research questions about the origins and development of creeping crises. In addition, we formulated questions on the convergence of pace and attention, and the prospects of managing these creeping crises. We introduce these questions below.

### What Determines the Development of Crisis?

Most scholars agree that crises are the outcome of a process. This process is—following Turner (1978)—often referred to in terms of an incubation phase. Overall, there has been little attention for this incubation phase. Turner (1978) talked about the accumulation of energy, which, he hypothesized, is facilitated by lax organizational cultures (see also Weick & Sutcliffe, 2007). Perrow (1984) offered a more precise theory, which explains how complexity and tight coupling produce conditions for a crisis to emerge.

When we talk about the development of a crisis, we must pay attention to both the temporal (pace) and spatial (place) dimension. The temporal dimension refers to the speed of threat accumulation. Creeping crises do not necessarily follow a linear trajectory: they may be slow to emerge and demonstrate small bursts of acceleration, followed by periods of stasis or reversal. The spatial development refers to the locations (geographical but also the systems) in which a crisis asserts itself. The creeping crisis may jump from one locale to another, or manifest itself simultaneously in different systems or arenas.

The brings us to our first question: *Why can creeping crises persist and develop for relatively long time periods without erupting and reaching quick closure*? This question pertains to the driving factors of a creeping crisis and enquires into their underlying dynamics. It accepts that crises do not necessarily follow linear trajectories; pathways meander and may even double back. But we don't really know why and how crises develop, accelerate, stall, revert and, at some point, erupt.

This brings us to a second question: *Are there tipping points in this process of crisis development?* A tipping point marks the transition between gradual development and sudden escalation. In theory, a crisis may have multiple tipping points. Presumably, a crisis may also have a *final* tipping point (after which the crisis has spent all of its energy). But we do not know for sure.

A third research question pertains to the detection of creeping crises: *How can these creeping crises be identified before they become manifest*? Creeping crises typically develop under the radar. Part of the answer, as we will elaborate below, may be found in the (lacking) attention span of professional crisis watchers. But it appears that the early phases of these creeping crises, when damage potential is building, are not always easy to detect. What we need are indicators of escalation and damage potential (some researchers speak of “precursor events”).

### What Determines the Level of Attention for Creeping Crises?

A perennial research question in political science and public policy studies asks why the general public, the media, and politicians pay attention to some problems while ignoring so many others (Kingdon, 1984; Rochefort & Cobb, 1994). Many problems exist for a long, long time without ever reaching crisis proportions. Some problems have survived so many policymaking interventions that they are referred to in terms of “wicked problems” (Rittel & Webber, 1973; cf. Ansell & Bartenberger, 2017). There is, in fact, quite a literature on persistent policy problems (see also Table 3).

**Table 3:** Related Terms

Vulnerability = weak point of a system or organization
Wicked problem (Rittel & Webber, 1973) = widely recognized societal problem that policymakers find hard to solve (cf. Ansell & Bartenberger, 2017).
Risk society (Beck, 1992) = modern society produces and distributes new risks in ambiguous ways
Institutional crisis (Boin & ‘t Hart, 2000) = when an institution is confronted with a quick drop in legitimacy
Black Swan (Taleb, 2008) = known risk that is considered statistically unlikely to materialize

In policy studies, crises are often described as rare moments of convergence when almost everybody, however briefly, agrees on the importance of a certain event or development. The creeping crisis complicates this widely accepted idea of crisis as point of convergence. It has the potential to trigger convergence, but it has not attracted sufficient levels of attention so that we can state with reasonable certainty that a society is gripped by this or that particular problem.

The creeping crisis shares certain characteristics with the problem that is neglected by politicians and policymakers. At the same time, we know that a creeping crisis differs from a chronic problem such as poverty, inequality, congested roads, or budget shortages. To be sure, pundits and analysts frequently use the term crisis to draw attention to “their” problem. But the creeping crisis is a new problem, surrounded by uncertainty and pregnant with potential to mobilize an entire society by the threat of doing harm to people's well‐being or cherished values. This analytical distinction between a “wicked problem” and “creeping crisis” is thus more than splitting hairs.

This brings us to our first research question: *What explains the level of public and political attention for emerging threats?* This question has a corollary question: Why do citizens and politicians *ignore* a crisis? These questions suggest a search for psychological and social mechanisms that refocus individual and collective attention towards new and potentially troubling issues.

The second question is closely related to the first: *When and how does the level of attention escalate?* Here again we encounter the idea of a tipping point, where the accumulation of attention passes a certain threshold that spurs politicians into action. Attention is one thing, but what really counts is a response.

### What Causes Synchronicity Between Crisis Pace and Level of Attention?

What sets a creeping crisis apart from a “full‐blown” crisis, at least initially, is the lack of remedial action that allows a creeping crisis to continue its build‐up of damage potential. Lack of remedial action is a function (at least partially) of fragmented attention or the absence of *political* attention. In other words, we assume that a society that is beset by a shared notion of crisis will try to remedy that crisis.^3^

This brings us to our first research question: *Under what conditions and through which mechanisms is the pace of crisis development matched by a requisite level of political attention?* ^4^ We cannot assume that there is a linear relation between accumulating threat potential and rising political attention. Anecdotal evidence suggests that emerging threats may invite limited remedial action, which may effectively if only temporarily halt the development of threat potential, thus allowing professional and political attention to shift to more urgent problems at hand. This, in turn, suggests that the relation between crisis development and political attention can be described in terms of feedback “loops” that can spiral upwards or downwards.

This brings us to our second research question: *What explains the relation between crisis dynamics and political attention flows*? We are especially interested to study why this relation may reach self‐propelling features: this is the case when an escalation in crisis development spurs political attention, which, in turn, may fuel the crisis (through ill—fated interventions, for instance). We also want to know how such cycles terminate or screech into reverse. Especially, after the creeping crisis has burst into view and political attention has peaked, it is intriguing to study how a “crisis in slow motion” wears out political attention spans, picking up speed again as societal and political stamina wanes.

### How to Manage Creeping Crises?

Creeping crises pose a unique combination of managerial challenges. It confronts policymakers with a complex problem that is not easily resolvable without the sustained attention of politicians. By the time political attention reaches a tipping point that enables concerted and urgent action, there is no longer just a complex problem to solve but a crisis to manage. Crisis management is hard enough for public managers (Boin, ‘t Hart, Stern, & Sundelius, 2016), but these challenges are compounded by the slow onset of the crisis: media and citizens will demand to know why this long‐coming crisis was not addressed earlier. The inevitable blame game will undermine the crisis management capacities of politicians that bear responsibility for the origins of the crisis (cf. Boin, Brown, & Richardson, 2019; Kuipers & Brandstrom, 2019).

This brings us to a set of research questions that are quite conventional in crisis management research but come with a special twist due to the creeping nature of these crises:

- *What determines administrative and political ownership of creeping crises?*
- *Why do detection and early warning not always lead to action? Is there something about the nature of creeping crises that militates against a heightened response?*
- *When, how, and under which conditions does ownership shift during the life cycle of a creeping crisis?*
- *How can a creeping crisis be arrested without a sense of political and/or social urgency?*
- *How can a creeping crisis be arrested without causing unintended consequences (creating the conditions for a bigger crisis down the road)?*
- *How can a creeping crisis that has manifested itself as an urgent threat be managed by politicians and/or public managers who are held responsible for their emergence?*
- *What are successful instances of creeping crisis management?*
- *Do creeping crises require different types of interventions than full‐blown crises?*

## Theoretical Perspectives

In this section, we briefly explore which powerful theories may get us started on the research questions posed above. We identify the most promising starting points, realizing and fully expecting that other theories will enter the picture upon further investigation. For now, this section serves to formulate a few initial insights to jump‐start the research on creeping crises.

### Theorizing on Crisis Development

The most relevant theories to serve as a starting point for our inquiry on crisis development focus on the complexity of sociotechnological systems (for an early statement, see LaPorte, 1975). Modern societies build technological systems that are increasingly hard to understand, even for those who design and operate the systems (examples include nuclear power plants, financial models, transport systems, and cyberspace). The consequence of this “designed ignorance” is that small glitches—errors, failures, or breakdowns—can develop into powerful threats. These small errors would be easy to fix or manage if the system operators would be aware of these “pathogens” (Turner, 1978). But the complexity of the system shields the malfunctioning, allowing it to persist and cause damage within the system (Perrow, 1984). This basic description of complex systems introduced a powerful insight: crises can, and often are, the outcome of an incubation process (Turner, 1978). The idea of incubation is, of course, temporal at heart.

By introducing the concept of tight coupling, Perrow (1984) added another dimension to the temporal understanding of crisis development. Perrow observed that many complex systems also display a high level of interdependencies between critical components of a system. The implication is that the consequences of a small incident or error may travel through interconnected systems. Just as a person who is infected with a communicable disease may spread the virus by entering hubs in a travel system, a technological error may trigger a chain of events in a complex system. The *incubation* concept is thus enriched with the concept of *escalation*: time and tight coupling may lead to unnoticed accumulation and acceleration of a crisis.

The study of complex sociotechnical systems has been boosted by the rise of the complexity perspective (Buchanan, 2000; Scheffer, 2009; Taylor, 2001). Originating in the study of physical and biological systems, this perspective lays the foundation for understanding the characteristics of a complex system as emergent from micro‐interactions within the system. Systems organize their own complexity, building up to a “tipping point” that brings a complex system to the edge of disaster. The idea of temporality is further enriched here by emphasizing the *non‐linearity* of the incubation phase (Ansell & Bartenberger, 2017). Crises incubate, develop, and escalate towards a tipping point—but the temporal dynamics can vary wildly during this process.

Complexity theory adds an intriguing paradox to our understanding of creeping crises. The tipping point in a creeping crisis process tends to mark a perfect state of equilibrium in the system: complexity is at its highest level, allowing for the highest functionality of that system. It is also the moment that a system may collapse. This, as we will see below, creates a serious dilemma for system managers.

The idea of complex systems producing their own crises has been further expanded with the concept of transboundary crises (Ansell, Boin, & Keller, 2010; Boin & Rhinard, 2008). When multiple complex systems are interwoven, a budding crisis may jump from one system to another. There are many examples of transboundary crises—threats that emerge because systems are closely connected. An electricity failure in one country can lead to a gas failure in another country. A problem with mortgages in the United States may undermine the common currency of the European Union. A virus originating in China may paralyze a city in Canada. Drought in Syria may trigger an immigration crisis in Europe. The transboundary perspective combines the notions of incubation, escalation, tipping points, and cascading effects.

#### Starting Insights

These theories, when read in conjunction, provide us with a set of starting insights with regard to the origins and development of creeping crises:

- Creeping crises may have long incubation periods, allowing them to accumulate threat potential in multiple systems.
- Creeping crises can begin their trajectory far away, their development and potential impact unnoticed by the managers of yet untouched systems.
- Creeping crises do not necessarily develop in a linear way; their development will see phases of slow accumulation and rapid escalation towards tipping points.
- Tipping points are critical moments for crisis management: they present the last moment for intervention, which, paradoxically, may mark the perfect state of the system.

### Theorizing on the Level of Attention

Political scientists have paid ample attention to the question why people—citizens, journalists, politicians—consider certain societal features problematic, even labeling them as threats or crises, when they ignore many other features and developments (which, objectively speaking, may carry much more damage potential than those on which attention is lavished). This question of attention is usually discussed in terms of “agendas.” These agendas are said to have limited “carrying capacity”—meaning that they only can hold so many problems deserving attention. The attention of the public, the media, and the political arena is inherently limited and selective. In addition, this attention also tends to be short‐lived: citizens, journalists, and politicians can only remain interested in a certain problem for so long (Downs, 1972).

Why do people focus their attention on one problem, ignoring others? Intriguingly, the characteristics of the problem at hand do not seem to matter much. People can worry about problems for which no evidence exists (UFOs and predatory clowns come to mind). They can blissfully ignore problems for which mountains of data exist, suggesting that disaster is imminent. Politicians in liberal democracies may choose to ignore certain problems, especially those that stretch into a future that exceeds their term.

To understand attention foci and cycles, we must, therefore, begin to treat problems as *social constructions*. This introduces us to the idea that various actors may work hard to push certain social constructions—we speak of “frames” in this context—because a certain frame serves their interest. The process of problem framing is not only a social process, it is political at heart. The process is also influenced by societal paradigms and fashions—influential ways of viewing problems and their effects on society. It is influenced by public institutions, which typically have a preference for certain problems (Schattschneider, 1960). All this suggests that the build‐up and convergence of attention—required to speak of a crisis—is a messy process. Two dominant theories can help us understand that process.

Kingdon's (1984) multiple streams theory describes the convergence of attention as a chaotic process. This theory posits that attention develops in different realms or “streams”: the public stream (what the public worries about), the policy stream (what policymakers think is important), and the political stream (what politicians want to decide on). The key idea in this perspective is that these streams are not tightly coupled. The public may worry about a certain problem, but that does not mean much if politicians ignore it (maybe because policymakers do not have a solution for it or because short‐term government cycles work against attention for long‐term problems). Something needs to happen to align public and political attention: a “focusing event.” This is an event that draws everybody's attention, even if it is for a brief moment in time. Kingdon emphasizes that actors can actively work to make that happen (successful actors are referred to as policy entrepreneurs) (see also Pralle, 2009).

An alternative and equally powerful theory has been formulated by Baumgartner and Jones (1993). They view attention as a political resource. Policy institutions canalize attention, jealously guarding politicians from people who seek to shift attention streams towards new or other problems (these people are described as the “losers” of institutionalized policy arenas). But these “losers” may work hard to draw attention to their cause. They can try to shift the “image” of the problem or they can bring the problem to the attention of other actors that were hitherto not involved (venue change). Their efforts may initiate an accumulation of attention, which is helped by events signaling that existing policy agendas are not helping to resolve problems that really matter. The accumulation of attention may reach a tipping point, after which the institutionalized agenda is discarded and a new agenda is adopted. This perspective shows intriguing similarities with the complexity perspective discussed above.

In addition to these policy theories, there are more specific theories that explain levels of attention with regard to emerging crises. Take, for instance, theories that explain the production of intelligence on threats. Noting that many wars came as a big surprise to political leaders (Pearl Harbor, Yom Kippur War), students of intelligence seek to understand why experts fail to read the signals of impending conflicts. These theories point to two additional factors. First, long‐standing paradigms may prevent policymakers and politicians from seeing the obvious (cf. Turner, 1978). Some threats may simply escape the imaginary capacity of policymakers and citizens alike (De Smet, Lagadec, & Leysen, 2012; cf. Boin et al., 2019). The Dutch did not expect the Germans to invade their country in 1940, because the Germans had allowed the Dutch to remain neutral during World War I. When the Germans invaded, it came as a shock (even though there was plenty of intelligence warnings about the impending invasion). Second, intelligence about impending attacks may be buried in (and concealed by) other messages (cf. Vaughan, 1996). Intelligence analysts may know but turn out to be poor communicators.

Yet another useful perspective comes from the field of security studies, which shows how governmental elites treat certain issues in exceptional, security‐laden ways in order to legitimize extraordinary (often extra‐legal) action (Williams, 2003). This is done not only through speech acts, as argued in the “Copenhagen School” approach, but also through everyday bureaucratic practices (Huysmans, 2011), the use of seemingly banal technology (Corry, 2012), and even visual imagery (Hansen, 2011). These approaches have much in common with the idea of crisis exploitation: politicians playing up a (potential) crisis to accomplish things that would be unthinkable or impossible under normal circumstances (Boin, McConnell, & ‘t Hart, 2008).

#### Starting Insights

These theories, when read in conjunction, provide us with a set of starting insights that help understand the level of attention for creeping crises:

- There are many creeping crises at any given time. Citizens, journalists, and politicians are likely to assess the various creeping crises in very different and fragmented ways.
- Without political attention, interventions seem unlikely to occur. Declaring a “climate emergency” did not help. Politicians remain hesitant.
- Without attention from the policymaking realm, creeping crises may long go undetected (no measurements‐>no problem).
- A creeping crisis does not become a full‐blown crisis until citizens, journalists, policymakers, and politicians recognize the “damage capacity” of the emerging threat and demand immediate remedial action.
- The characteristics of the threat cannot solely explain the level of attention for that threat. In modern societies, attention is unevenly and unequally distributed. There are always people trying to construe a condition or problem in terms of a crisis, but it takes a lot of effort (and some real fireworks) to make other people perceive a crisis. It takes time to arrive at a shared definition. Lack of solutions make that process more difficult (In the words of Wildavsky (1992): “no solution, no problem”).
- Institutions play a key role in the detection and labeling of creeping crises.
- A creeping crisis is an unrecognized crisis. As long as it is not widely recognized, politicians can stay away from the crisis, which keeps the crisis alive (and makes it harder to manage down the road).
- The general public may get worked up about a creeping crisis, but that may not last long. Following Downs (1972), we might say that the public becomes quickly bored with a crisis.
- Policymakers may recognize the problem but refrain from intervening as that might implicate them (they do not want to burn their hands).
- Ideological and cultural beliefs may feed political unwillingness or create “blind spots” (Turner, 1978).
- Creeping crisis may also provide opportunities for policy entrepreneurs to draw attention to their pet solution, indicate the potential damage of a problem when left unaddressed.

These insights add up to an important insight: a society may collectively recognize a creeping crisis but choose to ignore it. Societal dependency on the very conditions that produce the threat plays a role here. A society may not be willing to eliminate the threat‐producing conditions because many people depend on (or value) them—think of big data, cheap antibiotics, free movement, carbon‐emitting vehicles, and global communication. If the short‐term costs of elimination are too large to consider, a society may “decide” to live with the creeping crisis, treating only its occasional manifestations.

An additional insight pertains to the politics of crisis recognition. We wondered about the willingness of politicians to address creeping crises. These theories suggest different answers. They may not want to intervene. Attention for a creeping crisis may, after all, suggest previous neglect. They might want to intervene but don't know how to solve these creeping crises. Or the costs may be too high. For political purposes, it may simply be beneficial to play a potential crisis up or down.

### Theorizing on Synchronicity and Feedback Loops

The overarching core insight, thus far, may well be that a convergence between accumulating threat potential and growing political attention explains both the moment that a creeping crisis comes into the light as well as the window of opportunity that emerges to deal with the crisis. A creeping crisis can persist as long as society refuses to pay attention and address the underlying problem. The crisis label may, therefore, be necessary to mobilize the societal capacities required to adequately respond.

If a response is not organized in a proper and timely manner, the creeping crisis may continue to accumulate and play out in slow motion and full view. A negative feedback loop is then the likely result. Consider the following (rather simplified) example. Germany's history of antisemitism has caused an enduring level of shame among political elites. Confronted with the recent immigration crisis, it has been suggested that this sense of national shame informed the decision to allow unprecedented numbers of immigrants to enter the country. Unfortunately, some of these immigrants brought with them antisemitist attitudes. The immigration wave deepened the problem of antisemitism and boosted the political far‐right.

Very few theories have been formulated to understand the emergence and dynamics of feedback loops in governance systems. A good starting point is Kingdon's (1984) multiple streams theory, introduced above. Kingdon's framework offers a way to conceptualize interactions between streams that are marked by their own particular dynamics. It recognizes that actions or non‐actions in one stream may prompt a reaction in another, which then spurs development in a third, affecting the other two in return (and so on). It gives full play to the strategizing of key actors, who may well create unintended feedback loops. At the same time, this theory does not provide but a rudimentary beginning of these interaction loops.

#### Starting Insights

In lack of theoretical direction, we are left to formulate some elementary insights with regard to the convergence between attention and threat development:

- Manifestations of a creeping crisis may be viewed as the problem (rather than the indicator of an underlying problem). These manifestations may invite solutions that are both feasible and available, but only deal with short‐term effects (and do not address underlying factors). This will dial back public attention, but allow the crisis to persist.
- A build‐up of public attention may reach a tipping point after which political attention is mobilized.
- Without the crisis label, a large‐scale response that addresses root causes may not be considered necessary or politically feasible.

### Theorizing on the Management of Creeping Crises

We have no shortage on theories that explain the various stages and outcomes of crisis management. We have very few theories that focus on *creeping* crises. The relevant theories focus on what crisis scholars see as the key tasks of crisis management: detection, sense‐making, strategic decision making, and crisis communication. We will briefly discuss key theories with regard to each crisis management task and discuss how they pertain to creeping crises.

Crisis researchers tend to agree that the timely detection of crises is no easy task (Boin et al., 2016; Turner, 1978). They have identified a host of reasons. First, there are psychological factors that explain why people fail to recognize impending danger (Kahneman, 2012). These have to do with the inconceivability of certain unknown events: if you can't imagine a threat, you will not recognize it. Second, we know that many organizations are not designed to look for crises. Moreover, organizations do not adequately communicate relevant information with other relevant units (Turner, 1978). If we add to the mix the institutionalized ideas about what threats may be expected, we can understand why undefined threats pass through organizational filters. In hindsight, it is easy to see where the crisis originated and how it could have been spotted. The transboundary nature of modern societies make it harder to recognize creeping crises that manifest themselves in far‐away locales. The origins may be far away, but that “local” threat may extend itself in unforeseen ways through that very system.

The literature on so‐called High Reliability Organizations has identified best practices with regard to the early detection of potential risks (Rochlin, 1996; Weick & Sutcliffe, 2007), but it remains unclear if these practices work in most organizations.

Creeping crises pose an additional challenge (Boin & Lodge, 2019). Due to their ambiguous character, the “ownership” of these crises tends to be ill‐defined. We know that defined, agreed‐upon risks are monitored and addressed (through regulation, for instance) in the risk area. This area is dominated by professionals who are trained to minimize the chance that a known risk will materialize. When risks do unexpectedly materialize, their management is shifted to the crisis arena. Here, trained crisis professionals try to organize an effective and timely response. The problem with *creeping* crises is that they neither qualify as an agreed‐upon risk nor a full‐blown crisis. Without defined ownership, an organized response in the early phase is unlikely.

The crisis management literature has also paid ample attention to decision making processes during crises. A key challenge is the translation from ambiguous information to a strategic decision making agenda. If you don't know, exactly, what is going on, what decisions should be made? In the absence of verified knowledge, a rational problem‐solving approach cannot work. How, then, is crisis decision making informed? Crisis researchers make the point that crisis decision making is political at heart (Boin et al., 2016; ‘t Hart, 1993). One might expect that “politics” disappears in the background when a crisis or disaster emerges, but that rarely happens.

Due to the highly ambiguous nature of creeping crises, we expect political motives to play an important role in the decision making process. Research tells us that most politicians will seek to avoid the blame that may be assigned to them in the wake of a crisis (Boin et al., 2008; Hood, 2011). Their decision‐making calculations are informed by the probability that they will be cast as the villain during the aftermath of a crisis. The outcome of this calculation will inform their willingness to take ownership of the creeping crisis.

It follows from the above that any decisions on intervention, or lack thereof, will require an accompanying act of political communication. The framing of a crisis and the response to it significantly affect the public interpretation of the response and those who lead it. The legitimacy of public institutions and political leaders is at stake. And legitimacy is a key resource for leaders and institutions to mount an effective response. This all becomes a much more complex challenge in the case of a creeping crisis. As the nature of the threat is not widely agreed upon, the very act of branding it as a crisis (or not) will affect the reaction to that threat. It is easy to imagine how a botched meaning‐making process may undermine the legitimacy of leaders, which will undermine the effectiveness of the response. This, in turn, may further undermine legitimacy, prompting a vicious circle.

#### Starting Insights

The blossoming literature on crisis management provides us with a basic understanding of the challenges that crisis managers are likely to encounter. When we translate these understandings to the context of a creeping crisis, we arrive at the following starting insights:

- The recognition of, and response to a creeping crisis are political at heart. A rational problem‐solving approach will not suffice to understand why politicians do or don't react to a creeping crisis.
- The very act of recognizing a creeping crisis is likely to elevate societal attention for the threat. It also implies ownership, which may create a legitimacy problem down the road (if you own it, you broke it).
- Political leaders are highly motivated to avoid blame for the occurrence of a crisis. They may, therefore, seek to distance themselves from a creeping crisis, calculating that it will materialize after they are gone.

## Challenges for Practice

We seek to understand why lingering conditions with threat potential are not treated as a manageable risk or a full‐blown crisis. These creeping crises grow incrementally. They may erupt any moment, or so it seems to those who are paying attention. When the eruption actually happens, the creeping crisis is recognized in hindsight as a clear harbinger of things to come.

In explaining the emergence of crises, Turner (1978) used the analogy of pathogens lurking in a system, gathering strength and eventually paralyzing the system. The study of immunology both supports this analogy and provides clues to shape an approach towards understanding creeping crises (cf. Davis, 2018). The key in this approach is the search for anomalies: things that don't belong in the system. The body does it continually, using specially designed cells to search and destroy invaders.

We can use these insights to think about an approach towards dealing with creeping crises. That approach would consist of three steps:

1. Detect anomalies: in some cases this is easy [immigration, climate], in other cases it is not [financial systems, institutional crisis].
2. Assess whether the anomaly is dangerous: this is a political and social process in need of a clear method.
3. Neutralize the dangerous anomaly without causing undesired side effects.

The body uses T cells to detect, assess, and neutralize dangerous anomalies. In modern societies, institutions play the role of these T cells. Institutions contain societal sensors and filters that help to make sense of new developments, assessing whether developments are potentially dangerous, beneficial or both. Institutions embody societal values and time‐proven approaches. Institutions provide the key defenses against creeping crises, guarding against an overwrought response but also against an irresponsible *laissez‐fair* attitude.

Immunologists caution that the “on‐switch” of T cells can be problematic. That may be true for institutions as well. Typically, politicization plays a critical role. Politicization can switch an institution “on” or “off”. Sometimes it is easy [terrorism], often not: intervention can create many losers while the winners may yet have to be determined [climate change].

Immunologists prescribe resources for the effective functioning of T cells. The body needs sufficient sleep and the right food. Stress, bad habits, and diseases may undermine the effectiveness of T cells. In similar vein, we may say that a society needs to dedicate resources to “societal immunology”. This requires political capital. In its absence, infections can linger and proliferate.

### Implications for Practice

It seems obvious that the earlier a creeping crisis is officially recognized, the sooner an effective response can be organized. If nothing is done, the crisis festers, builds momentum, may change shape. Yet, intervening too soon may not be necessary. To borrow again from immunologists, we may view these small manifestations as pathogens: if conquered, they make society stronger. If left alone, the body may eventually become immune. An early intervention also carries risks: if draconic measures are introduced while the impact of the crisis is still uncertain, the issuing government may lose the public's trust quickly.

The challenge, then, is to detect which potential threat carries threat potential (so many potential crises out there) and has already caused damage. Those who are tasked with detection do not enjoy the benefits of hindsight. They likely are looking for something they have never seen before. They may have no idea where it comes from (risks emerging from one domain might not be understood in another domain).

Moreover, once detected, it is likely unclear what trajectory the creeping crisis will follow, which critical thresholds it may encounter, and what damage to expect. These people will have to create prospective meaning about a possible end state based on one or more small manifestations of the creeping crisis.

These insights leave policymakers with a set of options from which it may be hard to choose. They may elect to monitor the threat (pushing it into the risk domain). They may seek a middle position: monitor and contain, hoping that this will suffice to shift attention away from the threat. They may ignore the threat and wait if it blows over. After all, many problems tend to solve themselves. Or they may define a threat as a crisis, initiating a full‐press response. This latter option creates the dilemma of early intervention: action may work (great) but the cure may be worse than the disease. By treating the creeping crisis, the existence of a potential crisis has been acknowledged and ownership (success or failure) has been established.

In addition to timing, the hardest challenge may well be the design of the response. It is not clear what, if anything, is needed, what will work and who is best qualified to do it. But something must happen. After all, the very idea of a creeping crisis has real repercussions for accountability: apparently there was a sustained time period during which remedial action was possible. If the creeping crisis materializes, incumbent politicians lose. If it is not brought under control, the system loses.

A creeping crisis can expose the limitations of governance. The deficiencies of standing crisis management arrangements are laid bare in, for instance, the fight against pandemics, climate change, cyber threats, and disinformation campaigns. Governments seem unprepared to deal with crises that do not crystallize in sudden outbursts (plane crashes and factory explosions) but intertwine with deeper afflictions that corrode the environment, society, and world around us. All this prompts the question whether contemporary crisis management arrangements are a proper fit for the creeping crisis.

## Notes

1 The authors wish to thank the reviewers of this paper for their constructive comments. They thank the participants of the Stockholm workshop “Detecting Creeping Crisis: Mapping Challenges and Designing Strategies” (7–8 November 2019). They also thank Chris Ansell for his comments on an earlier version of this article. This research is funded by MSB.

2 This roughly follows the stage model of disaster as used by, for instance, the US Federal Emergency Management Agency.

3 We realize that this assumption may not be correct under all circumstances. But we think it works as a starting assumption.

4 This question presupposes the answer to the question what, exactly, would be a “sufficient” level of attention to guarantee an intervention. This subquestion requires attention as well.
